# Blood-based biomarkers and plasma Aβ assays in the differential diagnosis of Alzheimer’s disease and behavioral-variant frontotemporal dementia

**DOI:** 10.1186/s13195-024-01647-w

**Published:** 2024-12-30

**Authors:** Pablo Mohaupt, Jana Kindermans, Jérôme Vialaret, Sarah Anderl-Straub, Leonie Werner, Sylvain Lehmann, Christophe Hirtz, Markus Otto, Patrick Oeckl

**Affiliations:** 1 LBPC-PPC, Université de Montpellier, IRMB CHU de Montpellier, INM INSERM, Montpellier, France; 2 Department of Neurology, Ulm University Hospital, 89081 Ulm, Germany; 3 University Clinic and Polyclinic for Neurology, Martin-Luther-University Halle-Wittenberg, 06120 Halle (Saale), Germany; 4 German Center for Neurodegenerative Diseases (DZNE), Ulm, Germany

**Keywords:** Alzheimer’s disease, Frontotemporal lobar degeneration, Differential diagnosis, Amyloid-beta, Blood biomarker, Dementia, Mass spectrometry

## Abstract

**Introduction:**

The differentiation between Alzheimer’s disease (AD) and behavioral-variant frontotemporal dementia (bvFTD) can be complicated in the initial phase by shared symptoms and pathophysiological traits. Nevertheless, advancements in understanding AD’s diverse pathobiology suggest the potential for establishing blood-based methods for differential diagnosis.

**Methods:**

We devised a novel assay combining immunoprecipitation and mass spectrometry (IP-MS) to quantify Amyloid-beta (Aβ) peptides in plasma. We then assessed its performance against existing assays (Shimadzu and Simoa) and evaluated a range of other blood-based biomarkers, including GFAP, NfL, and pTau-181, for differentiating between AD and bvFTD.

**Results:**

The novel IP-MS assay measuring the Aβ42/40 ratio demonstrated an AUC of 0.82 for differentiating AD from control subjects. While it did not significantly outperform the composite biomarker score from the Shimadzu assay (AUC = 0.79, *P* = 0.67), it significantly outperformed the Shimadzu Aβ42/40 ratio (AUC = 0.65, *P* = 0.037) and the Simoa Aβ42/40 assay (AUC = 0.57, *P* = 0.023). Aβ biomarkers provided limited utility in distinguishing AD from bvFTD. In contrast, pTau181 and GFAP exhibited strong discriminatory power for differentiating AD from bvFTD, with AUCs of 0.90 and 0.87, respectively. Combining pTau181 and GFAP enhanced diagnostic accuracy, achieving an AUC of 0.94.

**Conclusion:**

We introduced a novel IP-MS assay that demonstrated comparable precision to the Shimadzu composite score in differentiating AD from non-neurodegenerative control groups. However, Aβ levels did not enhance the discrimination between AD and bvFTD. Furthermore, our findings support the utility of combining pTau181 and GFAP as a robust strategy for the blood-based differentiation of AD and bvFTD.

**Supplementary Information:**

The online version contains supplementary material available at 10.1186/s13195-024-01647-w.

## Introduction

Alzheimer’s disease (AD) manifests as a mixed proteinopathy in which Amyloid pathology and tau-pathology work in concert to induce cognitive decline [1]. Recent advances have substantially enhanced our understanding of AD’s complex and varied pathobiology [2]. This progress has led to the identification of specific pathobiological traits and their corresponding biofluid markers. Furthermore, technological innovations have enabled the quantification of these biomarkers in blood, expanding beyond their initial discovery in cerebrospinal fluid (CSF) [3]. Despite these advancements, differentiating AD from bvFTD remains a significant clinical challenge, largely due to their overlapping symptoms and pathophysiological traits. bvFTD is part of the broader FTLD spectrum, which encompasses diverse underlying pathologies, including tauopathies and TDP-43 proteinopathies, further complicating accurate diagnosis.

The focus in AD diagnostics is increasingly on tau protein with its phosphorylated amino acid residues, which arise as crucial biomarkers for blood-based diagnosis [4–6]. Emerging evidence suggests that tau protein with site-specific phosphorylation can specifically correlate with either amyloid pathology or tau-pathology [7–10]. However, the exact mechanisms underlying these associations remain elusive. Furthermore, elevated levels of phosphorylated tau have been correlated with renal dysfunction, a connection not observed with the amyloid-beta (Aβ) 42/40 ratio [11, 12]. This distinction underscores the complex interplay between tau and amyloid pathologies in AD. Considering potential confounders such as patients with comorbidities like kidney disease, the use of a panel of biomarkers may better control factors affecting individual biomarker classes. The direct relationship between the Aβ42/40 ratio and the formation of amyloid plaques is well-documented, reinforcing the significance of these biomarkers in understanding and diagnosing AD. The initial efforts to clinically validate blood-based assays for the measurement of Aβ peptides faced considerable obstacles, primarily due to their low abundance and propensity to adhere to surfaces, which limited the effectiveness of early enzyme-linked immunosorbent assays (ELISA). Recent advancements have led to the development of several assays for quantifying Aβ peptides, notably through IP-MS with mass spectrometry and through immunoassays utilizing the Single Molecule Array (Simoa) platform [13–16]. These assays offer a promising avenue for assessing amyloid load in AD brain, thereby playing a pivotal role in the preliminary screening of clinical trial participants. This development is particularly significant given that individuals with different manifestations of amyloid and tau pathologies may exhibit varied responses to specific therapeutic interventions. Furthermore, amyloid pathology is, excluding cases presenting with AD comorbidity, conspicuously absent in syndromes of the frontotemporal lobar degeneration (FTLD) disease spectrum. Consequently, these assays hold potential for facilitating the differentiation between AD and FTLD cases.

In this study we extend our analysis beyond the conventional focus on phosphorylated tau. In addition to amyloid pathology, aberrations between AD and FTLD are observed in inflammatory responses of reactive astrocytes, and in the level of axonal damage. These anomalies can be tracked through the quantification of Glial fibrillary acidic protein (GFAP) and Neurofilament light protein (NfL) respectively. Our research evaluates the diagnostic performance of plasma Aβ assays alongside other blood-based biomarkers for the differential diagnosis of AD and FTLD. Specifically, we compare the outcomes of three distinct Aβ assays conducted on various platforms: (1) immunoprecipitation-mass spectrometry (IP-MS) with Matrix-Assisted Laser Desorption/Ionization-Time of Flight (MALDI-TOF) detection, (2) an immunoassay utilizing the Simoa platform, and (3) a newly-developed IP-MS assay with Electrospray Ionization-Multiple Reaction Monitoring (ESI-MRM). Additionally, we evaluate the comparative effectiveness of these assays alongside other available blood-based biomarkers, including NfL, GFAP, and pTau-181, in distinguishing AD from NNC and bvFTD.

## Methods

### Study participants

Participants were recruited at the Department of Neurology, Ulm University Hospital, within the German FTLD consortium (www.ftld.de), a quality-controlled, monitored, multicentre initiative. The study cohort comprised individuals with clinically diagnosed Alzheimer’s disease (AD; *n* = 18) and behavioral-variant frontotemporal dementia (bvFTD; *n* = 20), alongside non-neurodegenerative controls (NNC). All participants underwent a standardized neurological and neuropsychological examination, along with brain MRI. Cognitive function was assessed using the Mini-Mental State Examination (MMSE). The NNC group included individuals with normal clinical and cognitive assessments who did not meet criteria for dementia or other neurological or psychiatric disorders. The diagnosis of bvFTD was established according to Rascovsky et al., while AD diagnosis was confirmed according to McKhann [17, 18]. Differential diagnosis of AD and bvFTD was supported by CSF core AD biomarkers (Aβ42, pTau181, t-Tau) but not for all patients. bvFTD participants were negative for amyloid pathology. CSF (lumbar puncture) and EDTA plasma were collected, centrifuged and stored within two hours at -80 °C. All individuals or their legal proxies provided written informed consent for inclusion into this study and it was approved by the ethics committee of Ulm University (approval number 39/11). Demographic details of the participants are summarized in Table 1.

**Table 1:** Demographic characteristics of patient samples

Demographic characteristics	AD	NNC	bvFTD	*P*-value
*N*	18	12	20	
Age at sampling (years)	67 [61–73]	71 [59–74]	63 [59–67]	0.266
Sex (male/female)	11/7	5/7	13/7	0.409
CSF Aβ42 (pg/mL)	536 [469–569] (*n* = 11)	-	1072 [908–1154] (*n* = 20)	< 0.0001
CSF t-tau (pg/mL)	830 [617–1136] (*n* = 11)	-	324 [283–514] (*n* = 20)	< 0.001
CSF p-tau181 (pg/mL)	111 [63–164] (*n* = 11)	-	54 [39–60] (*n* = 20)	< 0.05
MMSE	19 [16–25] (*n* = 15)	29 [28–30] (*n* = 11)	25 [23.5–27] (*n* = 19)	< 0.0001
Plasma GFAP (pg/mL)	282 [186–377]	130 [96–191]	127 [105–164]	< 0.001
Plasma NfL (pg/mL)	31.5 [21.9–38.8]	15.2 [13.4–23.9]	51.7 [28.2–77.8]	< 0.01
Plasma pTau-181 (pg/mL)	2.11 [1.84–2.64]	1.17 [1.02–1.31]	0.98 [0.78–1.26]	< 0.0001
Plasma Aβ40 Simoa, (pg/mL)	222 [201–254] (*n* = 16)	231 [211–314] (*n* = 12)	206 [178–235] (*n* = 19)	0.097
Plasma Aβ40 Shimadzu (pg/mL)	8.01 [6.27–8.84]	8.11 [6.82–9.23]	7.34 [6.60 − 8.54]	0.723
Plasma Aβ40 Ulm (pg/mL)	455 [410–484]	452 [423–573]	452 [400–487]	0.671
Plasma Aβ42 Simoa (pg/mL)	8.94 [8.03–9.79] (*n* = 16)	10.0 [8.96–12.9] (*n* = 12)	9.19 [7.95–10.8] (*n* = 19)	0.313
Plasma Aβ42 Shimadzu (pg/mL)	0.35 [0.32–0.41]	0.38 [0.38–0.44]	0.36 [0.32–0.39]	0.079
Plasma Aβ42 Ulm (pg/mL)	58.8 [54.9–64.0]	68.1 [63.0–82.5]	57.7 [53.6–65.6]	< 0.05
Plasma Aβ42/40 Simoa	0.039 [0.036–0.041] (*n* = 16)	0.040 [0.037–0.047] (*n* = 12)	0.046 [0.042–0.051] (*n* = 19)	< 0.05
Plasma Aβ42/40 Shimadzu	0.044 [0.042–0.051]	0.048 [0.045–0.055]	0.048 [0.042 − 0.052	0.372
Plasma Aβ42/40 Ulm	0.131 [0.123–0.138]	0.145 [0.139 − 0.167]	0.130 [0.125–0.142]	< 0.01
Shimadzu composite score	0.719 [0.028–1.12]	-0.126 [-0.473–0.136]	0.281 [-0.222–0.661]	< 0.05

Note: Demographics are expressed as median values along with their interquartile ranges. For the analysis of continuous variables, the Kruskal-Wallis test was applied to assess disparities among the groups. Furthermore, the chi-square test for goodness of fit was employed for the examination of categorical variables. *P*-values derived from these analyses are provided within the table for reference and interpretive purposes. The number of samples is indicated per group in the demographic characteristics, or stated within the cell if aberrant

### Determination of AD core biomarkers in CSF

CSF levels of Aβ42, pTau181 and t-Tau were measured by ELISA (Fujirebio, Gent, Belgium) during diagnostic workup of patients in the routine CSF laboratory of the Department of Neurology, Ulm University Hospital, according to local SOPs and under regular quality control.

### IP-MS measurement of Aβ38, Aβ40 and Aβ42 in plasma samples (Ulm)

EDTA plasma samples, calibrators and QC samples (490μL each) were mixed with ^15^N-Aβ38, ^15^N-Aβ40 and ^15^N-Aβ42 (rPeptide, Watkinsville, GA, USA) as internal standards, with triethylammonium bicarbonate (TEAB, final 120mM) and Tween 20 (final 0.05%). Magnetic beads (0.5 mg per sample, Thermo 14302D) covalently coupled with 6E10 antibody (Biolegend, 2 μg/mg beads) were added to each sample and incubated on a rotator over night at 4 °C. Beads were washed three times with 500μL 50mM TEAB/0.1% n-Dodecyl-β-D-maltoside and eluted with 25μL 50mM glycine HCl (pH 2.5). The eluted Aβ peptides were digested with 10μL of a TrypN working solution (12.5ng/μL, Protifi, Fairport, NY, USA) for 2.5 h at 37 °C and stopped with 10μL of 0.5% TFA in acetonitrile and stored in the autosampler at 4 °C. A volume of 20μL was injected into a QTRAP6500 mass spectrometer (Sciex) coupled to an Eksigent MicroLC200 and Agilent 1260 pump. Peptides were loaded on an Acclaim PepMap100, C18 trap column (5 μm, 0.3 × 5 mm, Thermo) using mobile phase A: 0.05% TFA and mobile phase B: 90% acetonitrile, 0.1% ammonium hydroxide and a flow rate of 200μL/min. Separation of peptides was performed on a HALO Fused-Core C18, 100 × 0.5 mm analytical column (Eksigent, Framingham, MA, USA) at 60 °C and a gradient time of 9.85 min (5–35%B, total run time 17.5 min) with mobile A: 4% DMSO, 0.1% formic acid and mobile phase B: 4% DMSO, 96% acetonitrile, 0.1% formic acid. Peptides were infused into the QTRAP6500 mass spectrometer by electrospray ionization and measured in MRM mode using the following transitions: Aβ38 (aa28-38, 508.3→784.5 (b8+), 508.3→883.5 (b9+), 508.3→653.4 (b7+)); Aβ40 (aa28-40, 607.4→997.6 (b11+), 607.4→548.8 (b12++), 607.4→499.3 (b11++)); Aβ42 (aa28-42, 699.4→598.4 (b13++), 699.4→548.8 (b12++), 699.4→1096.7 (b12+)). Data were analysed using Skyline software v23.1 and for quantification, external calibration curves were generated using the light-to-heavy peak area ratios of calibrator samples and a quadratic function with 1/x² weighting. Calibrator samples (8-point calibration) were prepared in a surrogate matrix (3% bovine serum albumin in PBS) using synthetic Aβ38, Aβ40 and Aβ42 (Sigma) in the range of 1-100pg/mL (Aβ38, Aβ42) and 10-1000pg/mL (Aβ40). Plasma QC samples were included in all runs to monitor performance of measurements. The method was validated in terms of intraassay (1.2–10.5%) and interassay CV (3.4–7.4%), dilution stability (tested for 2- and 4-fold dilution, accuracy 89.9-110.7%), spike-in recovery (20pg/mL for Aβ38 and Aβ42, 200pg for Aβ40, recovery 93.1-100.4%) and stability at room temperature for 2 h and up to 3 freeze-thaw-cycles (accuracy 80.2-108.1%). Intraassay CV of QC samples during measurement of patient samples was 1.2–10.5%.

### IP-MALDI-MS measurement of Aβ40, Aβ42 and composite biomarker in plasma samples (Shimadzu)

Measurements of Aβ peptides, Aβ40 and Aβ42, along with the amyloid precursor protein fragment APP669-711, were performed at The Centre Hospitalier Universitaire (CHU) of Montpellier. Quantification was achieved using MALDI-TOF MS (AXIMA Assurance, Shimadzu) following dual IP, as previously described. Briefly, the IP utilized Dynabeads M‐270 Epoxy as the solid phase, coated with the mouse monoclonal antibody 6E10. A composite biomarker was then derived by calculating the mean Z-score of the ratios Aβ40/Aβ42 and APP669‐711/Aβ42.

### Determination of Aβ40, Aβ42, GFAP, pTau181 and NfL in plasma samples by Simoa and Ella

Simoa measurements were performed with a HD-1 analyzer in Ulm. Aβ40 and Aβ42 were measured in plasma samples using the Neurology 3-plex A (N3PA) assay (Quanterix, Billerica, MA, USA) according to the manufacturer’s instructions. Intraassay CV of plasma QC samples was 2.5–5.5%. GFAP was measured with the Simoa GFAP Discovery Kit (intraassay CV 3.4%) and pTau181 with the pTau181 Advantage V2 Kit (intraassay CV 14.5%). NfL was measured in plasma samples with the Ella automated microfluidic immunoassay (Ella Human NF-L Kit from ProteinSimple, San Jose, CA, USA) (intraassay CV 18.7%).

### Data analysis

Graphical analyses were conducted in R (version 4.3.0). Sample size estimation was based on a power analysis of the composite biomarker score from the IP-MS assay (Shimadzu) using a previously published dataset [14]. We assumed a moderate effect size to justify a power of 0.80 and an alpha level of 0.05, estimating a minimum sample size of 13 to detect differences between AD and control groups. Scatterplots were generated using ggplot2, and Spearman’s rank correlation assessed inter-assay correlations. Levene’s test evaluated homogeneity of variances, and Shapiro-Wilk tests of raw and log-transformed residuals assessed normality. Group comparisons were performed with rank-based ANCOVA adjusted for age and sex, followed by Mann-Whitney U tests with Bonferroni correction for post hoc analysis. Receiver Operating Characteristic (ROC) analysis was used to assess classification performance. Statistical comparisons of AUCs between assays were performed using the DeLong test.

## Results

### Comparison of three plasma Aβ assays

**Fig. 1:**
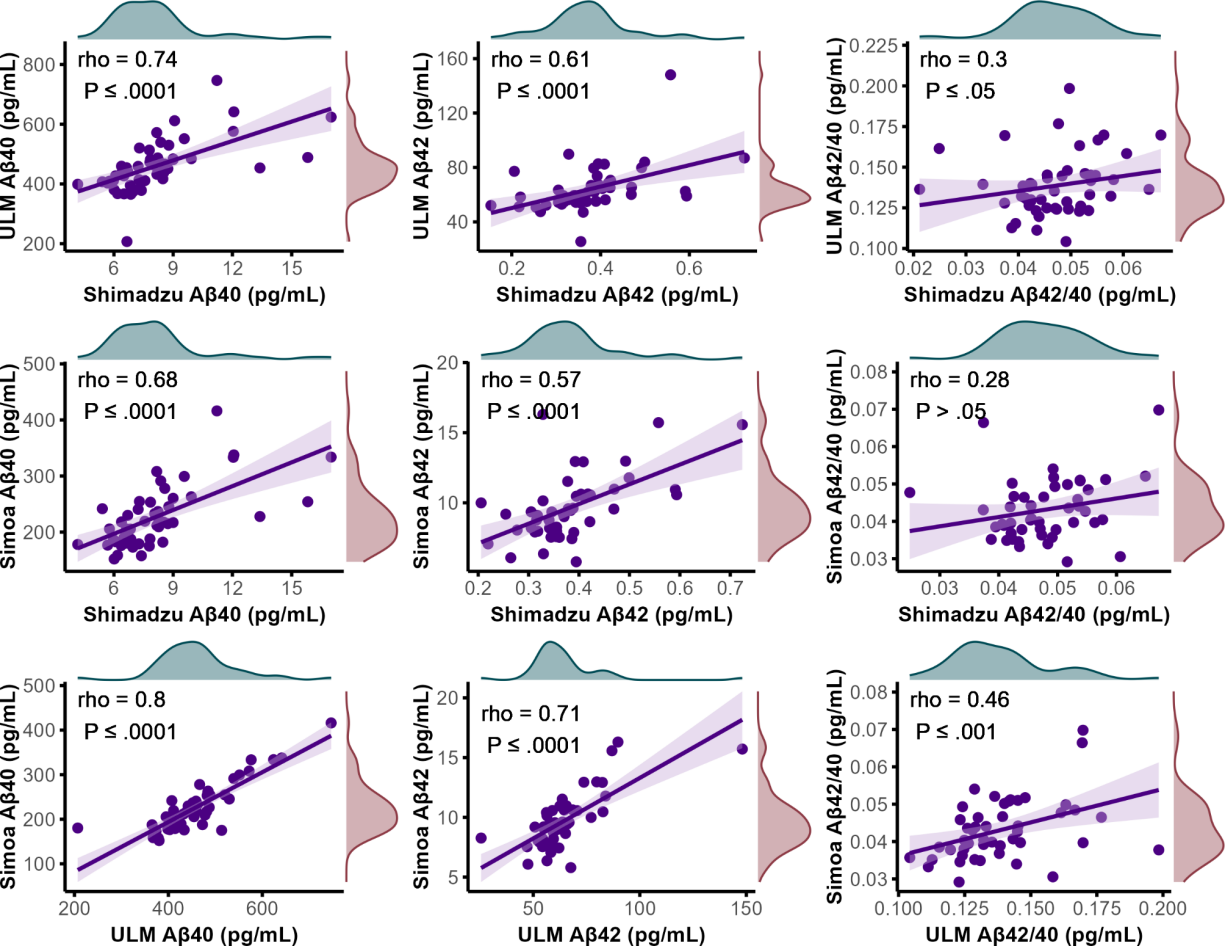
Scatter plots visualizing the correlations between plasma concentrations of Aβ40 and Aβ42, along with the Aβ42/Aβ40 ratio, across various assays. Each point represents data from a single clinical sample. Concentrations of Aβ40 and Aβ42 were determined using three different assays: Shimadzu, Simoa, and the newly developed Ulm assay. A regression line in purple indicates the overall trend, with lighter purple shading denoting the confidence interval. The analysis employs Spearman’s rank correlation to calculate correlation coefficients, highlighting the relationships among these assays. Marginal density plots along the x (teal) and y axes (red) illustrate the distribution of the data for each variable

A strong correlation was observed across the Aβ40 concentrations in patient samples, as determined by various assays (Fig. 1). The novel assay dubbed “ULM” executed with IP-MS utilizing ESI-MRM, showed a good correlation, with Spearman’s rho values of 0.74 (*P* ≤ 0.0001) with the Shimadzu assay, and a correlation of 0.80 (*P* ≤ 0.0001) with the assay executed on the Simoa platform. A moderate to good correlation was observed in Aβ 42 concentrations, with Spearman’s rho values of 0.61 (*P* ≤ 0.0001) with the Shimadzu assay, and a correlation of 0.71 (*P* ≤ 0.0001) with the assay executed on the Simoa platform. Only a weak correlation was observed in the Aβ42/40 ratio between the ULM assay and the Shimadzu assay, with a Spearman’s rho of 0.30 (*P* ≤ 0.05), and a weak to moderate correlation of 0.46 (*P* ≤ 0.001) with the Aβ42/40 ratio on the Simoa platform. Comparatively, the Shimadzu assay’s correlations with the Simoa platform were modest but slightly lower, with Spearman’s rho values of 0.68 (*P* ≤ 0.0001) for Aβ40, 0.57 (*P* ≤ 0.0001) for Aβ42, and 0.28 with for the Aβ42/40 ratio. However, the latter was above the threshold of statistical significance with *P* > 0.05.

### Group-wise comparison for differential diagnosis

**Fig. 2:**
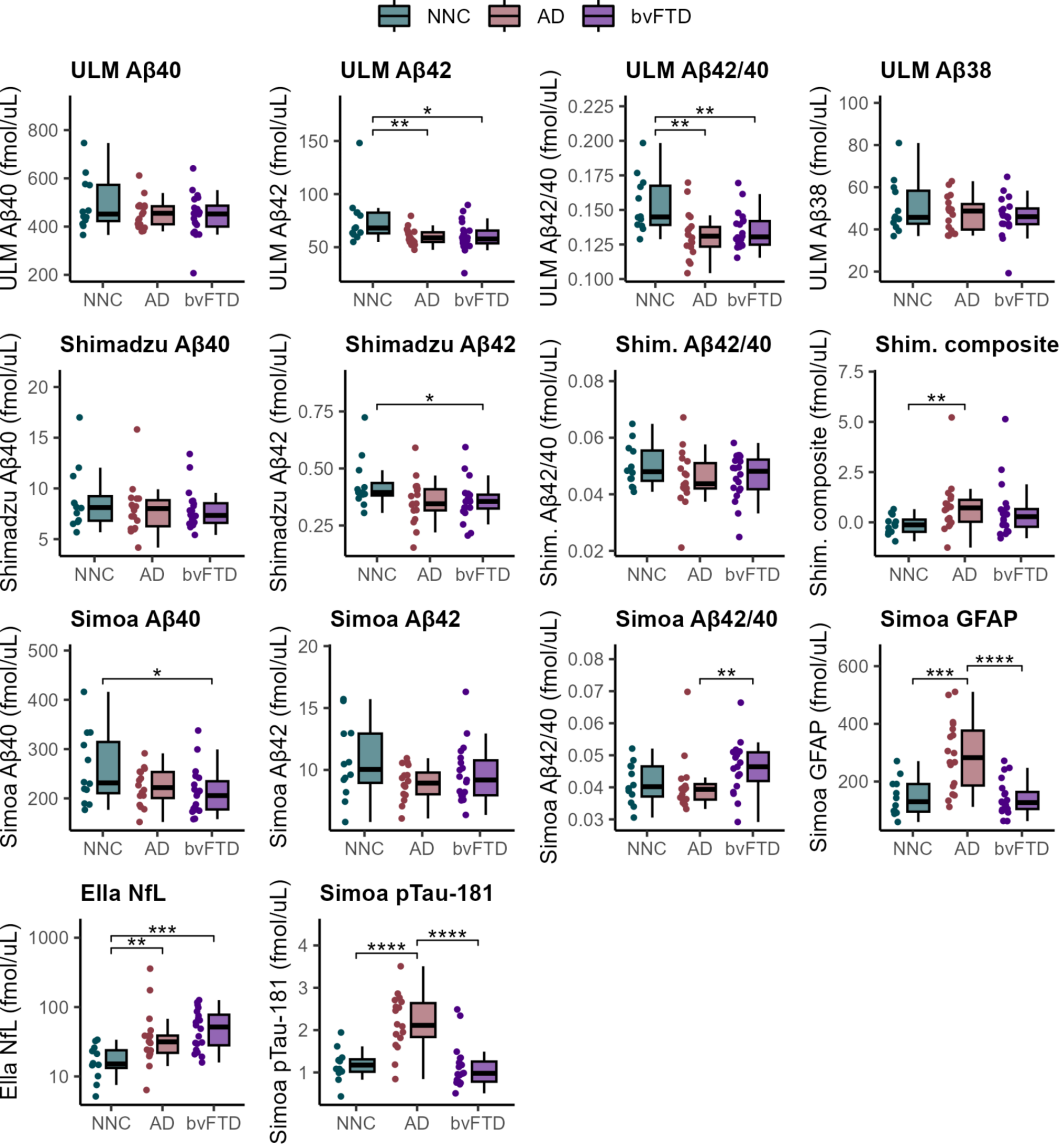
Comparative analysis of blood-based biomarker levels in clinical samples individuals with Alzheimer’s disease (AD, *n* = 18), behavioral-variant frontotemporal dementia (bvFTD, *n* = 20), or non-neurodegenerative controls (NNC, *n* = 12). The distribution of biomarker levels across groups is visualized using boxplots overlaid with dot plots, showing both the interquartile range and individual data point distribution. Statistical comparisons were conducted using rank-based ANCOVA to assess overall differences among groups, with age and gender as covariates. Pairwise Mann-Whitney U tests with Bonferroni correction for multiple comparisons were performed for post-hoc analyses. Significance levels are denoted as follows: *P ≤ 0.05, **P ≤ 0.01, ***P ≤ 0.001, and ****P ≤ 0.0001

We conducted group-wise comparisons of plasma biomarkers using boxplots (Fig. 2) to evaluate their ability to distinguish between patient groups for differential diagnosis. The Simoa assay did not achieve statistical significance in differentiating AD patients from NNC. However, it demonstrated significant discrimination between AD and bvFTD (*P* < 0.01) based on the Aβ42/40 ratio. The Shimadzu assay reached statistical significance in differentiating AD and NNC only when using the composite biomarker score (*P* < 0.01). In contrast, the ULM assay exhibited robust discrimination capabilities, achieving significance in differentiating AD patients from NNC using both Aβ42 and the Aβ42/40 ratio (*P* < 0.01, and *P* < 0.01, respectively). Both measures were also significant in distinguishing bvFTD from NNC (*P* < 0.05, and *P* < 0.01, respectively).

Established blood-based biomarkers, including GFAP, NfL, and pTau181, demonstrated superior differentiation capabilities across groups. GFAP effectively distinguished AD from bvFTD and NNC (*P* < 0.0001 and *P* < 0.001, respectively). NfL achieved significance in differentiating bvFTD and AD from NNC (*P* < 0.001 and *P* < 0.01, respectively), while pTau181 distinguished AD from both bvFTD and NNC with high statistical significance (*P* < 0.0001 for both comparisons).

### Classification based on plasma Aβ and blood-based biomarkers

**Fig. 3:**
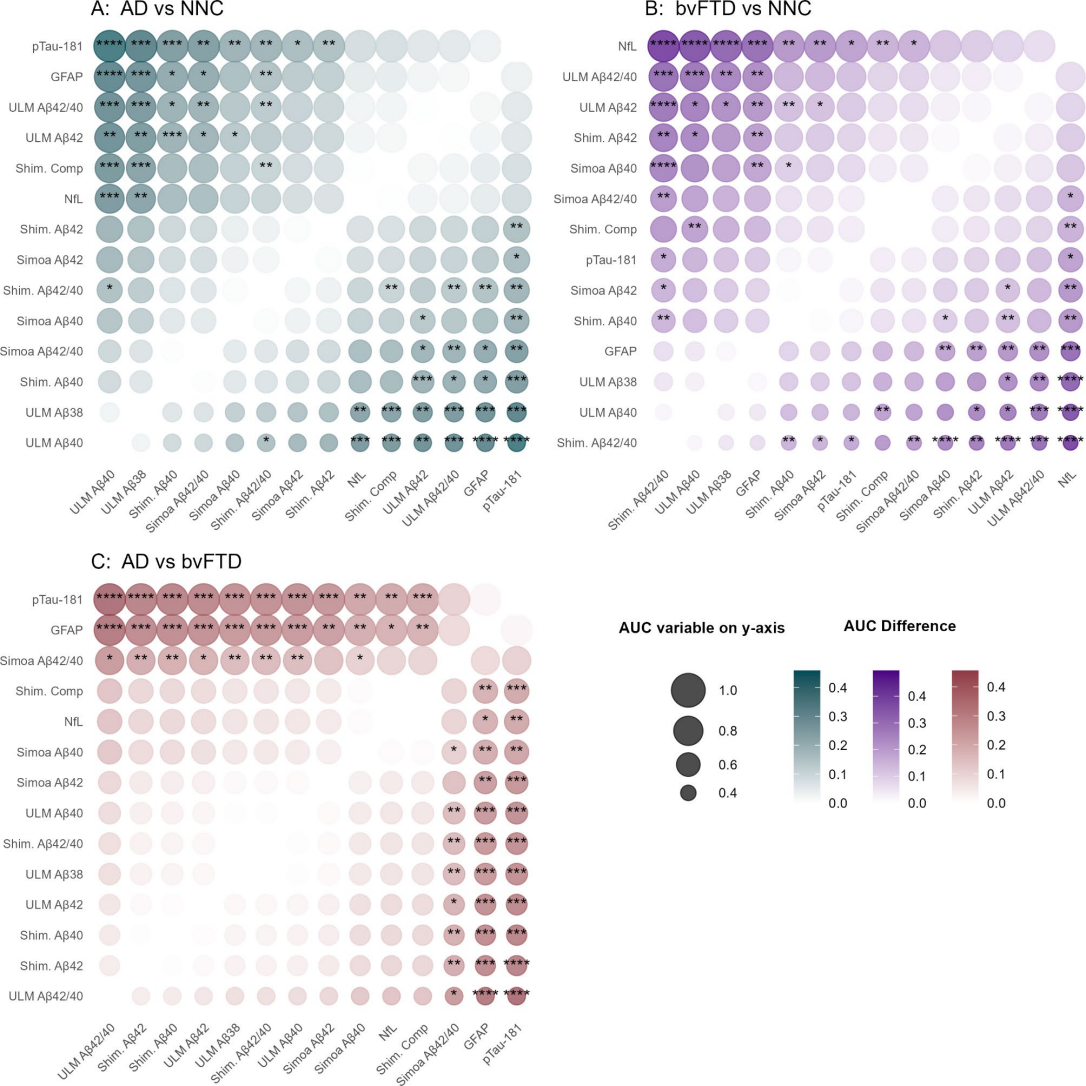
Comparison of the area under the curve (AUC) values of blood-based assays and biomarkers across three pairwise group comparisons: (**A**) Alzheimer’s disease (AD) vs. non-neurodegenerative controls (NNC), (**B**) behavioral variant frontotemporal dementia (bvFTD) vs. NNC, and (**C**) AD vs. bvFTD. The AUC for each biomarker was calculated from ROC analyses. Pairwise AUC comparisons were performed using the DeLong test. Each panel presents a heatmap showing AUC differences between biomarkers, with the point size reflecting the AUC value of the biomarker on the y-axis. The color intensity represents the absolute AUC difference between the biomarker on the y-axis and the corresponding biomarker on the x-axis. Statistically significant differences are labeled with significance levels: ***, **, and *, corresponding to *p*-values of ≤ 0.001, ≤ 0.01, and ≤ 0.05, respectively. Biomarkers are arranged by AUC from highest to lowest on the y-axis

We conducted ROC analysis to assess the classification performance of biomarkers and assays (Fig. 3; Table 2). Comprehensive ROC analyses, including DeLong’s test comparisons, are presented in Supplementary Tables 1–3. In the discrimination of AD from NNC, pTau181 demonstrated a notable AUC of 0.90 (95% CI: 0.78–1.00), followed by GFAP (AUC = 0.85, 95% CI: 0.72–0.99). The Aβ42/40 ratio and Aβ42 concentration, both measured using the ULM Assay, and the composite score derived from the Shimadzu assay, displayed AUCs of 0.82 (95%CI = 0.67–0.97), 0.82 (95%CI = 0.67–0.97), and 0.79 (95%CI = 0.63–0.95), respectively. The ULM Aβ42/40 assay (AUC = 0.82) did not show a statistically significant difference from the Shimadzu composite score (AUC = 0.79; *p* = 0.67). However, it significantly outperformed both the Shimadzu Aβ42/40 assay (AUC = 0.65; *p* = 0.037) and the Simoa Aβ42/40 assay (AUC = 0.57; *p* = 0.023). The Shimadzu composite score also displayed a higher, though non-significant, AUC than the Simoa assay (*p* = 0.11). In distinguishing AD from bvFTD both pTau181 and GFAP retained prominence, yielding an AUC of 0.90 (95%CI = 0.79–1.00) and 0.87 (95%CI = 0.76–0.98), respectively. A lower classification efficacy to differentiate AD from bvFTD instances was observed for Aβ assays and NfL, with only Aβ42/40 measured with Simoa reaching an AUC of 0.76 (95%CI = 0.58–0.93). Furthermore, NfL was most effective in differentiating bvFTD from NNC groups, with an AUC of 0.88 (95%CI = 0.77–1.00).

**Table 2:** Diagnostic accuracy of Aβ40, Aβ42, Aβ42/40 ratio across assays, NfL, GFAP, and p-tau181

AD vs. NNC	*n*	AUC	95% CI	Sensitivity	Specificity
Aβ40 Simoa	16/12	0.64	[ 0.41–0.86 ]	0.50	0.75
Aβ40 Shimadzu	18/12	0.56	[ 0.34–0.78 ]	0.72	0.42
Aβ40 Ulm	18/12	0.44	[ 0.21–0.67 ]	0.44	0.67
Aβ42 Simoa	16/12	0.68	[ 0.45–0.90 ]	0.75	0.58
Aβ42 Shimadzu	18/12	0.69	[ 0.49–0.88 ]	0.67	0.75
Aβ42 Ulm	18/12	0.82	[ 0.67–0.97 ]	0.67	0.83
Aβ42/40 Simoa	16/12	0.57	[ 0.33–0.80 ]	0.75	0.50
Aβ42/40 Shimadzu	18/12	0.65	[ 0.45–0.85 ]	0.56	0.75
Aβ42/40 Ulm	18/12	0.82	[ 0.67–0.97 ]	0.78	0.83
Aβ composite score Shimadzu	18/12	0.79	[ 0.63–0.95 ]	0.72	0.75
Plasma GFAP	18/12	0.85	[ 0.72–0.99 ]	0.72	0.83
Plasma NfL	18/12	0.79	[ 0.78–1.00 ]	0.72	0.75
Plasma pTau181	18/12	0.90	[ 0.78–1.00 ]	0.83	0.92

bvFTD vs. NNC	n	AUC	95% CI	Sensitivity	Specificity
Aβ40 Simoa	19/12	0.72	[ 0.54–0.91 ]	0.63	0.75
Aβ40 Shimadzu	20/12	0.59	[ 0.38–0.80 ]	0.65	0.58
Aβ40 Ulm	20/12	0.41	[ 0.20–0.63 ]	0.55	0.5
Aβ42 Simoa	19/12	0.60	[ 0.37–0.82 ]	0.53	0.75
Aβ42 Shimadzu	20/12	0.74	[ 0.56–0.93 ]	0.75	0.75
Aβ42 Ulm	20/12	0.77	[ 0.60–0.93 ]	0.65	0.83
Aβ42/40 Simoa	19/12	0.67	[ 0.47–0.87 ]	0.74	0.58
Aβ42/40 Shimadzu	20/12	0.39	[ 0.18–0.60 ]	0.5	0.58
Aβ42/40 Ulm	20/12	0.79	[ 0.63–0.95 ]	0.8	0.67
Aβ composite score Shimadzu	20/12	0.67	[ 0.48–0.86 ]	0.6	0.75
Plasma GFAP	20/12	0.48	[ 0.25–0.70 ]	0.65	0.5
Plasma NfL	20/12	0.88	[ 0.77–1.00 ]	0.75	0.83
Plasma pTau181	20/12	0.62	[ 0.41–0.83 ]	0.55	0.83

AD vs. bvFTD	n	AUC	95% CI	Sensitivity	Specificity
Aβ40 Simoa	16/19	0.61	[ 0.42–0.81 ]	0.62	0.58
Aβ40 Shimadzu	18/20	0.51	[ 0.31–0.70 ]	0.56	0.65
Aβ40 Ulm	18/20	0.55	[ 0.36–0.74 ]	0.44	0.75
Aβ42 Simoa	16/19	0.56	[ 0.37–0.76 ]	0.75	0.42
Aβ42 Shimadzu	18/20	0.50	[ 0.31–0.69 ]	0.56	0.60
Aβ42 Ulm	18/20	0.52	[ 0.33–0.71 ]	0.61	0.50
Aβ42/40 Simoa	16/19	0.76	[ 0.58–0.93 ]	0.88	0.74
Aβ42/40 Shimadzu	18/20	0.54	[ 0.35–0.73 ]	0.56	0.65
Aβ42/40 Ulm	18/20	0.44	[ 0.25–0.63 ]	0.50	0.55
Aβ composite score Shimadzu	18/20	0.62	[ 0.44–0.81 ]	0.61	0.70
Plasma GFAP	18/20	0.87	[ 0.76–0.98 ]	0.78	0.80
Plasma NfL	18/20	0.62	[ 0.44–0.81 ]	0.83	0.55
Plasma pTau181	18/20	0.90	[ 0.79–1.00 ]	0.89	0.90

### Classification of AD and bvFTD instances by biomarker combinations

**Fig. 4:**
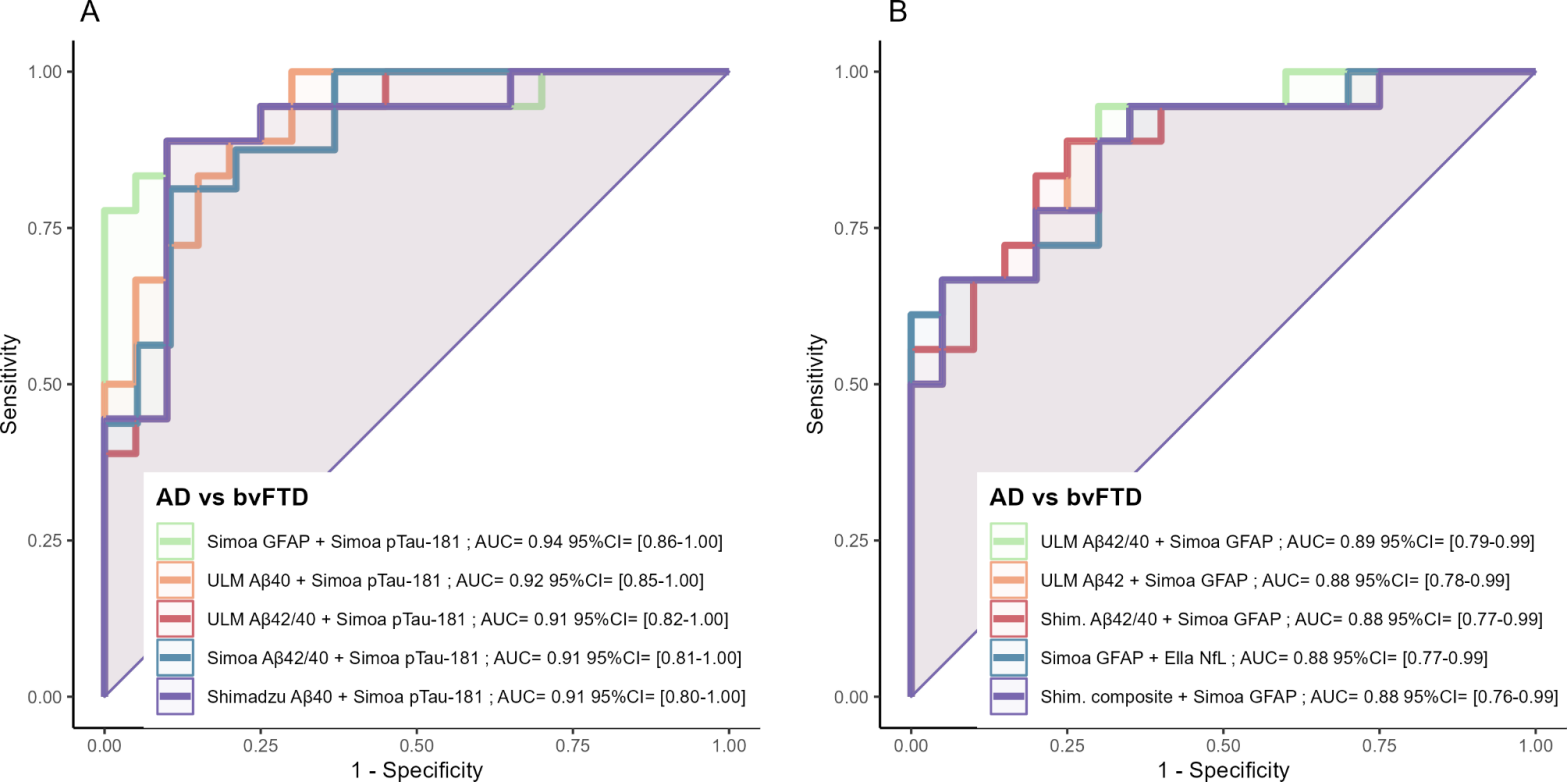
Receiver operating characteristic (ROC) curves illustrating the diagnostic performance of two-biomarker combinations in distinguishing Alzheimer’s disease (AD) from behavioral variant frontotemporal dementia (bvFTD). AUC values were derived from logistic regression models for all possible two-biomarker combinations, including all assays and biomarkers in present study. The top five combinations, ranked by AUC, are presented. The analysis contrasts models with (**A**) and without (**B**) the inclusion of pTau-181. It should be noted that none of the biomarker combinations were significantly better than their individual counterparts, according to DeLong’s test

Logistic regression models were used to evaluate whether combinations of biomarkers could improve classification efficacy between AD and bvFTD cases. The highest AUC was obtained with the combination of pTau181 and GFAP (AUC = 0.94, 95%CI: 0.86–1.00) (Fig. 4), but no notable improvements were observed when adding either Aβ42, Aβ40, or Aβ42/40 to the equations. The same observation was made in models without pTau181. However, comparative analysis using DeLong’s test for ROC curves revealed that none of the biomarker combinations significantly outperformed their individual counterparts. This outcome may reflect the limited statistical power due to the small sample size.

## Discussion

In this study, we compared a large panel of blood biomarkers to determine their effectiveness in the differential diagnosis of AD and bvFTD. This evaluation included a novel IP-MS assay for the quantification of Aβ peptides in plasma. Plasma pTau181 and GFAP showed the best diagnostic performance, which was further enhanced when combined. Conversely, NfL and Aβ peptides demonstrated limited diagnostic value. In comparison to existing Aβ assays (Shimadzu, Simoa), our novel IP-MS Aβ assay (Ulm) demonstrated comparable performance to the Shimadzu composite score, which includes an additional peptide alongside Aβ42 and Aβ40. Both assays effectively distinguished AD patients from NNC subjects. The novel assay outperformed both the Shimadzu Aβ42/40 ratio and the Simoa Aβ42/40 assay. No significant difference was observed between AD and bvFTD in our patient cohort using any of these assays.

Our findings contribute to a growing body of literature that investigates the role of plasma Aβ in neurodegenerative diseases beyond AD. For instance, a study exploring Aβ levels in AD, FTD, and DLB (*n* = 160), showed a significant decrease in Aβ42/40 ratios in AD and FTD cases, which aligns with our observations [14]. Similarly, the latter study and a study examining Aβ42/40 ratios in mild cognitive impairment (MCI), AD, and conditions within the FTLD spectrum (*n* = 362), identified no distinct diagnostic advantage of Aβ42/40 ratios in differentiating AD from FTD [19]. This contrasts with another study (*n* = 300), which did report a significant difference in Aβ42/40 ratios measured with Simoa between AD, controls, and FTD cases [20]. Notably, our results obtained through the Simoa assay were significant for differentiating AD from bvFTD cases too, but not when compared to controls. However, comparison and interpretation of studies investigating Aβ peptides in blood is difficult because of strong differences in the performance of reported Aβ assays. Comparative studies indicated that mass spectrometry-based assays perform best and this is also evident from our study showing better diagnostic performance of the Shimadzu and Ulm assays to discriminate AD from controls than the Simoa assay [21, 22]. These findings underscore the complexity of diagnosing neurodegenerative dementias based on plasma Aβ measurements and highlight the variability in assay outcomes across different platforms. Nevertheless, the novel ULM assay demonstrates promising performance in differentiating AD from controls, warranting further investigation.

Plasma pTau181 and GFAP exhibited significant potential for differentiating between AD and bvFTD. This is in agreement with other studies from our and other groups and further strengthens their utility to be used in clinical routine settings [19, 23, 24]. Notably, the differentiation accuracy did increase when pTau181 was combined with GFAP in a logistic regression model, although not significant. Recently, a study involving a prospective memory cohort of 385 individuals utilized a blend of pTau181, GFAP, NfL, and APOE genotype to distinguish between FTD from non-FTD cases, achieving an AUC of 0.87 [25]. Further, in a neuropathology cohort (*n* = 316), assessments of NfL, pTau181, and GFAP distinguished AD from FTD with notable accuracy (AUCs of 0.79, 0.96, and 0.81, respectively) [26]. No notable improvement was observed in our study when logistic regression models were applied to Aβ42/40 with either pTau181, GFAP, or NfL. Thus, our study especially supports the combination of pTau181 with GFAP. The combination of GFAP with other pTau variants (e.g. pTau217) might also further improve their diagnostic performance and specificity and should be investigated in future studies. In contrast to pTau181 and GFAP, NfL was not identified as a dependable biomarker for distinguishing between these conditions. This can be attributed to the increase of blood NfL levels during both conditions and, thus, a lack of specificity which is already known from previous studies [27].

Other than phosphorylation, recent studies describe specific tau-related aberrations unique to AD. Specifically, serum brain-derived tau was specifically increased in AD and was able to differentiate AD from bvFTD (AUC = 0.93) [28]. Further investigations highlighted distinctive variations in the abundance of the third and fourth repeats in the microtubule-binding region (MTBR) of tau in AD compared to FTLD, suggesting AD-specific tau abnormalities beyond phosphorylation and their potential as biomarkers differentiating AD from primary tauopathies [29]. Combination of this novel tau variants with GFAP might further improve their differential diagnostic potential which should be addressed in future studies.

### Limitations

A notable limitation of our study is the relatively small cohort size, as well as the absence of CSF biomarker data for some participants in both the AD and NNC groups. A power analysis was conducted based on the composite biomarker score from the benchmark IP-MS assay to determine an adequate sample size for detecting differences between AD and control groups. However, this power analysis did not extend to our newly developed assay, and thus the study may still be underpowered to detect smaller effects specific to the new assay. The focus of our study was on evaluating the combined performance of assays and biomarkers in differentiating AD and bvFTD, prioritizing the breadth of initial assessments over replication. Due to the specialized nature of this study and the resources required, particularly samples from paticipants with bvFTD, and analysis carried out over different laboratories, a replication study was not feasible within the scope of this project. Furthermore, this study did not explore the correlation between plasma Aβ42/40 and CSF Aβ42/40 ratios or the accumulation of amyloid plaques in the brain. This correlation is a vital determinant in evaluating the accuracy of assays in reflecting cerebral amyloid pathology. It is also worth noting that our measurements with the Simoa assay were conducted using the 3-plex configuration. However, comparative analyses in existing literature suggest that the 4-plex Simoa assay is more effective for determining Aβ42/40 ratios in plasma [30]. Therefore, incorporating the 4-plex Simoa assay might have offered a more appropriate comparison for our purposes.

## Conclusions

In summary, we introduced an IP-MS assay for Aβ42/40 that demonstrates comparable accuracy to the Shimadzu composite score in distinguishing AD from control groups. The novel assay outperformed both the Shimadzu Aβ42/40 ratio and the Simoa Aβ42/40 assay. Our findings suggest that Aβ levels alone do not provide additional diagnostic value in differentiating AD from bvFTD. Notably, the synergistic use of pTau181 and GFAP emerges as a potent combination for the blood-based differential diagnosis of AD and bvFTD.

## Electronic Supplementary Material

Below is the link to the electronic supplementary material.

Supplementary Material 1

## Data Availability

The dataset analyzed during the current study is available from the corresponding author on reasonable request.

## Abbreviations

Aβ: Amyloid-beta
AD: Alzheimer’s disease
bvFTD: Behavioral-variant frontotemporal dementia
CSF: Cerebrospinal fluid
ELISA: Enzyme-linked immunosorbent assays
EMA: European Medicines Agency
ESI-MRM: Electrospray Ionization-Multiple Reaction Monitoring
FDA: Food and Drug Administration
FTLD: Frontotemporal lobar degeneration
GFAP: Glial fibrillary acidic protein
IP-MS: Immunoprecipitation-mass spectrometry
MALDI-TOF: Matrix-Assisted Laser Desorption/Ionization-Time of Flight
MCI: Mild cognitive impairment
MMSE: Mini-Mental State Examination
MTBR: Microtubule-binding region
NfL: Neurofilament light protein
NNC: Non-neurodegenerative control
pTau-181: Tau protein with phosphorylation at the 181st amino acid residue
pTau-205: Tau protein with phosphorylation at the 205th amino acid residue
pTau-217: Tau protein with phosphorylation at the 217th amino acid residue
ROC: Receiver Operating Characteristic
Simoa: Single Molecule Array
TEAB: Triethylammonium bicarbonate

## References

[1] Hardy JA, Higgins GA. Alzheimer’s disease: the amyloid cascade hypothesis. Science. 1992;256:184–5. 1566067 10.1126/science.1566067

[2] Scheltens P, De Strooper B, Kivipelto M, Holstege H, Chételat G, Teunissen CE, et al. Alzheimer’s disease. Lancet. 2021;397:1577–90. 33667416 10.1016/S0140-6736(20)32205-4 PMC8354300

[3] Teunissen CE, Verberk IMW, Thijssen EH, Vermunt L, Hansson O, Zetterberg H, et al. Blood-based biomarkers for Alzheimer’s disease: towards clinical implementation. Lancet Neurol. 2022;21:66–77. 34838239 10.1016/S1474-4422(21)00361-6

[4] Ashton NJ, Brum WS, Di Molfetta G, Benedet AL, Arslan B, Jonaitis E et al. Diagnostic accuracy of a plasma phosphorylated Tau 217 immunoassay for Alzheimer disease pathology. JAMA Neurology. 2024 [cited 2024 Feb 2]; 10.1001/jamaneurol.2023.5319 10.1001/jamaneurol.2023.5319 PMC10804282 38252443

[5] Barthélemy NR, Bateman RJ, Hirtz C, Marin P, Becher F, Sato C, et al. Cerebrospinal fluid phospho-tau T217 outperforms T181 as a biomarker for the differential diagnosis of Alzheimer’s disease and PET amyloid-positive patient identification. Alzheimers Res Ther. 2020;12:26. 32183883 10.1186/s13195-020-00596-4 PMC7079453

[6] Lehmann S, Schraen-Maschke S, Vidal J-S, Delaby C, Buée L, Blanc F et al. Clinical value of plasma ALZpath pTau217 immunoassay in the assessment of mild cognitive impairment. medRxiv; 2024 [cited 2024 Feb 2]:2024.01.21.24301570. https://www.medrxiv.org/content/10.1101/2024.01.21.24301570v1

[7] Mattsson-Carlgren N, Janelidze S, Bateman RJ, Smith R, Stomrud E, Serrano GE, et al. Soluble P‐tau217 reflects amyloid and tau pathology and mediates the association of amyloid with tau. EMBO Mol Med. 2021;13:e14022. 33949133 10.15252/emmm.202114022 PMC8185545

[8] Therriault J, Vermeiren M, Servaes S, Tissot C, Ashton NJ, Benedet AL, et al. Association of phosphorylated tau biomarkers with amyloid positron emission tomography vs Tau positron emission tomography. JAMA Neurol. 2023;80:188–99. 36508198 10.1001/jamaneurol.2022.4485 PMC9856704

[9] Barthélemy NR, Saef B, Li Y, Gordon BA, He Y, Horie K, et al. CSF tau phosphorylation occupancies at T217 and T205 represent improved biomarkers of amyloid and tau pathology in Alzheimer’s disease. Nat Aging. 2023;3:391–401. 37117788 10.1038/s43587-023-00380-7 PMC10154225

[10] Lantero-Rodriguez J, Montoliu-Gaya L, Benedet AL, Vrillon A, Dumurgier J, Cognat E, et al. CSF p-tau205: a biomarker of tau pathology in Alzheimer’s disease. Acta Neuropathol. 2024;147:12. 38184490 10.1007/s00401-023-02659-w PMC10771353

[11] Bellomo G, Bayoumy S, Megaro A, Toja A, Nardi G, Gaetani L et al. Fully automated measurement of plasma Aβ42/40 and p-tau181: Analytical robustness and concordance with cerebrospinal fluid profile along the Alzheimer’s disease continuum in two independent cohorts. Alzheimer’s & Dementia. [cited 2024 Feb 13];n/a. https://onlinelibrary.wiley.com/doi/abs/10.1002/alz.13687 10.1002/alz.13687 PMC11032583 38323780

[12] Lehmann S, Schraen-Maschke S, Vidal J-S, Allinquant B, Bombois S, Gabelle A, et al. Plasma Aβ42/Aβ40 ratio is independent of renal function. Alzheimer’s Dement. 2023;19:2737–9. 36774628 10.1002/alz.12949

[13] Nakamura A, Kaneko N, Villemagne VL, Kato T, Doecke J, Doré V, et al. High performance plasma amyloid-β biomarkers for Alzheimer’s disease. Nature. 2018;554:249–54. 29420472 10.1038/nature25456

[14] Thijssen EH, Verberk IMW, Kindermans J, Abramian A, Vanbrabant J, Ball AJ et al. Differential diagnostic performance of a panel of plasma biomarkers for different types of dementia. Alzheimer’s & Dementia: Diagnosis, Assessment & Disease Monitoring. 2022 [cited 2024 Feb 14];14. https://www.ncbi.nlm.nih.gov/pmc/articles/PMC9107685/10.1002/dad2.12285 PMC9107685 35603139

[15] Alcolea D, Delaby C, Muñoz L, Torres S, Estellés T, Zhu N, et al. Use of plasma biomarkers for AT(N) classification of neurodegenerative dementias. J Neurol Neurosurg Psychiatry. 2021;92:1206–14. 34103344 10.1136/jnnp-2021-326603

[16] Hirtz C, Busto GU, Bennys K, Kindermans J, Navucet S, Tiers L, et al. Comparison of ultrasensitive and mass spectrometry quantification of blood-based amyloid biomarkers for Alzheimer’s disease diagnosis in a memory clinic cohort. Alzheimer’s Res Therapy. 2023;15:34. 10.1186/s13195-023-01188-8 PMC9938625 36800984

[17] Rascovsky K, Hodges JR, Knopman D, Mendez MF, Kramer JH, Neuhaus J, et al. Sensitivity of revised diagnostic criteria for the behavioural variant of frontotemporal dementia. Brain. 2011;134:2456–77. 21810890 10.1093/brain/awr179 PMC3170532

[18] McKhann GM, Knopman DS, Chertkow H, Hyman BT, Jack CR, Kawas CH, et al. The diagnosis of dementia due to Alzheimer’s disease: recommendations from the National Institute on Aging-Alzheimer’s Association workgroups on diagnostic guidelines for Alzheimer’s disease. Alzheimers Dement. 2011;7:263–9. 21514250 10.1016/j.jalz.2011.03.005 PMC3312024

[19] Thijssen EH, La Joie R, Wolf A, Strom A, Wang P, Iaccarino L, et al. Diagnostic value of plasma phosphorylated tau181 in Alzheimer’s disease and frontotemporal lobar degeneration. Nat Med. 2020;26:387–97. 32123386 10.1038/s41591-020-0762-2 PMC7101073

[20] Chouliaras L, Thomas A, Malpetti M, Donaghy P, Kane J, Mak E, et al. Differential levels of plasma biomarkers of neurodegeneration in Lewy body dementia, Alzheimer’s disease, frontotemporal dementia and progressive supranuclear palsy. J Neurol Neurosurg Psychiatry. 2022;93:651–8. 35078917 10.1136/jnnp-2021-327788 PMC9148982

[21] Zicha S, Bateman RJ, Shaw LM, Zetterberg H, Bannon AW, Horton WA et al. Comparative analytical performance of multiple plasma Aβ42 and Aβ40 assays and their ability to predict positron emission tomography amyloid positivity. Alzheimers Dement. 2022. 2023;19:956–66. 10.1002/alz.12697 PMC10518222 35820077

[22] Janelidze S, Bali D, Ashton NJ, Barthélemy NR, Vanbrabant J, Stoops E et al. Head-to-head comparison of 10 plasma phospho-tau assays in prodromal Alzheimer’s disease. Brain. 2023;146:1592–601. 10.1093/brain/awac333 PMC10115176 36087307

[23] Oeckl P, Halbgebauer S, Anderl-Straub S, Steinacker P, Huss AM, Neugebauer H, et al. Glial fibrillary acidic protein in serum is increased in Alzheimer’s disease and correlates with cognitive impairment. J Alzheimers Dis. 2019;67:481–8. 30594925 10.3233/JAD-180325

[24] Oeckl P, Anderl-Straub S, Von Arnim CAF, Baldeiras I, Diehl-Schmid J, Grimmer T et al. Serum GFAP differentiates Alzheimer’s disease from frontotemporal dementia and predicts MCI-to-dementia conversion. J Neurol Neurosurg Psychiatry. 2022;93:659–67. 10.1136/jnnp-2021-328547 35477892

[25] Sarto J, Ruiz-García R, Guillén N, Ramos-Campoy Ó, Falgàs N, Esteller D, et al. Diagnostic performance and clinical applicability of blood-based biomarkers in a prospective memory clinic cohort. Neurology. 2023;100:e860–73. 36450604 10.1212/WNL.0000000000201597 PMC9984216

[26] Baiardi S, Quadalti C, Mammana A, Dellavalle S, Zenesini C, Sambati L, et al. Diagnostic value of plasma p-tau181, NfL, and GFAP in a clinical setting cohort of prevalent neurodegenerative dementias. Alzheimers Res Ther. 2022;14:153. 36221099 10.1186/s13195-022-01093-6 PMC9555092

[27] Oeckl P, Anderl-Straub S, Danek A, Diehl-Schmid J, Fassbender K, Fliessbach K, et al. Relationship of serum beta-synuclein with blood biomarkers and brain atrophy. Alzheimer’s Dement. 2023;19:1358–71. 36129098 10.1002/alz.12790

[28] Gonzalez-Ortiz F, Turton M, Kac PR, Smirnov D, Premi E, Ghidoni R, et al. Brain-derived tau: a novel blood-based biomarker for Alzheimer’s disease-type neurodegeneration. Brain. 2022;146:1152–65. 10.1093/brain/awac407 PMC9976981 36572122

[29] Horie K, Barthélemy NR, Spina S, VandeVrede L, He Y, Paterson RW, et al. CSF tau microtubule-binding region identifies pathological changes in primary tauopathies. Nat Med. 2022;28:2547–54. 36424467 10.1038/s41591-022-02075-9 PMC9800273

[30] Wojdała AL, Bellomo G, Toja A, Gaetani L, Parnetti L, Chiasserini D. CSF and plasma Aβ42/40 across Alzheimer’s disease continuum: comparison of two ultrasensitive Simoa^®^ assays targeting distinct amyloid regions. Clin Chem Lab Med (CCLM). 2024;62:332–40. 37656487 10.1515/cclm-2023-0659

