# Supplementary material for: Blood-based biomarkers and plasma Aβ assays in the differential diagnosis of Alzheimer’s disease and behavioral-variant frontotemporal dementia
Source: Alzheimers Res Ther. 2024 Dec 30;16:279. doi: 10.1186/s13195-024-01647-w (PMC11687143; doi:10.1186/s13195-024-01647-w)
Supplement: Supplementary file 1 — Supplementary Material 1 [file 13195_2024_1647_MOESM1_ESM.docx]

**Supplementary table 1:** ROC analysis AD vs SCD

| Biomarker | AUC | AUC  Lower_CI | AUC  Upper_CI | Youden  Index | Sensitivity | Specificity | DeLong_Comparisons |
| --- | --- | --- | --- | --- | --- | --- | --- |
| ULM Aβ42/40 | 0.82 | 0.67 | 0.97 | 0.61 | 0.78 | 0.83 | ULM Aβ42 p = 0.96  ULM Aβ40 p = 0.0010  Shim. Aβ42/40 p = 0.037  Shim. Aβ42 p = 0.23  Shim. Aβ40 p = 0.065  Shim. Comp p = 0.67  GFAP p = 0.79  NfL p = 0.73  pTau-181 p = 0.47  Simoa Aβ40 p = 0.24  Simoa Aβ42 p = 0.28  Simoa Aβ42/40 p = 0.023  ULM Aβ38 p = 0.0031 |
| ULM Aβ42 | 0.82 | 0.67 | 0.97 | 0.53 | 0.61 | 0.92 | ULM Aβ42/40 p = 0.96  ULM Aβ40 p = 0.027  Shim. Aβ42/40 p = 0.2  Shim. Aβ42 p = 0.12  Shim. Aβ40 p = 0.0030  Shim. Comp p = 0.79  GFAP p = 0.78  NfL p = 0.79  pTau-181 p = 0.42  Simoa Aβ40 p = 0.051  Simoa Aβ42 p = 0.12  Simoa Aβ42/40 p = 0.059  ULM Aβ38 p = 0.038 |
| ULM Aβ40 | 0.44 | 0.21 | 0.67 | 0.11 | 0.44 | 0.67 | ULM Aβ42/40 p = 0.0010  ULM Aβ42 p = 0.027  Shim. Aβ42/40 p = 0.053  Shim. Aβ42 p = 0.17  Shim. Aβ40 p = 0.58  Shim. Comp p = 0.0037  GFAP p = 0.0004  NfL p = 0.0089  pTau-181 p = 0.0009  Simoa Aβ40 p = 0.38  Simoa Aβ42 p = 0.25  Simoa Aβ42/40 p = 0.34  ULM Aβ38 p = 0.54 |
| Shim. Aβ42/40 | 0.65 | 0.45 | 0.85 | 0.31 | 0.56 | 0.75 | ULM Aβ42/40 p = 0.037  ULM Aβ42 p = 0.2  ULM Aβ40 p = 0.053  Shim. Aβ42 p = 0.76  Shim. Aβ40 p = 0.64  Shim. Comp p = 0.04  GFAP p = 0.041  NfL p = 0.19  pTau-181 p = 0.017  Simoa Aβ40 p = 0.92  Simoa Aβ42 p = 0.72  Simoa Aβ42/40 p = 0.74  ULM Aβ38 p = 0.1 |
| Shim. Aβ42 | 0.69 | 0.49 | 0.88 | 0.42 | 0.67 | 0.75 | ULM Aβ42/40 p = 0.23  ULM Aβ42 p = 0.12  ULM Aβ40 p = 0.17  Shim. Aβ42/40 p = 0.76  Shim. Aβ40 p = 0.14  Shim. Comp p = 0.29  GFAP p = 0.19  NfL p = 0.47  pTau-181 p = 0.024  Simoa Aβ40 p = 0.8  Simoa Aβ42 p = 0.92  Simoa Aβ42/40 p = 0.49  ULM Aβ38 p = 0.24 |
| Shim. Aβ40 | 0.56 | 0.34 | 0.78 | 0.19 | 0.94 | 0.25 | ULM Aβ42/40 p = 0.065  ULM Aβ42 p = 0.0030  ULM Aβ40 p = 0.58  Shim. Aβ42/40 p = 0.64  Shim. Aβ42 p = 0.14  Shim. Comp p = 0.13  GFAP p = 0.051  NfL p = 0.15  pTau-181 p = 0.0062  Simoa Aβ40 p = 0.41  Simoa Aβ42 p = 0.3  Simoa Aβ42/40 p = 0.98  ULM Aβ38 p = 0.69 |
| Shim. Comp | 0.79 | 0.63 | 0.95 | 0.56 | 0.56 | 1 | ULM Aβ42/40 p = 0.67  ULM Aβ42 p = 0.79  ULM Aβ40 p = 0.0037  Shim. Aβ42/40 p = 0.04  Shim. Aβ42 p = 0.29  Shim. Aβ40 p = 0.13  GFAP p = 0.56  NfL p = 0.97  pTau-181 p = 0.19  Simoa Aβ40 p = 0.43  Simoa Aβ42 p = 0.51  Simoa Aβ42/40 p = 0.11  ULM Aβ38 p = 0.0097 |
| GFAP | 0.85 | 0.72 | 0.99 | 0.58 | 0.67 | 0.92 | ULM Aβ42/40 p = 0.79  ULM Aβ42 p = 0.78  ULM Aβ40 p = 0.0004  Shim. Aβ42/40 p = 0.041  Shim. Aβ42 p = 0.19  Shim. Aβ40 p = 0.051  Shim. Comp p = 0.56  NfL p = 0.41  pTau-181 p = 0.55  Simoa Aβ40 p = 0.2  Simoa Aβ42 p = 0.29  Simoa Aβ42/40 p = 0.057  ULM Aβ38 p = 0.0016 |
| NfL | 0.79 | 0.62 | 0.95 | 0.47 | 0.89 | 0.58 | ULM Aβ42/40 p = 0.73  ULM Aβ42 p = 0.79  ULM Aβ40 p = 0.0089  Shim. Aβ42/40 p = 0.19  Shim. Aβ42 p = 0.47  Shim. Aβ40 p = 0.15  Shim. Comp p = 0.97  GFAP p = 0.41  pTau-181 p = 0.23  Simoa Aβ40 p = 0.43  Simoa Aβ42 p = 0.6  Simoa Aβ42/40 p = 0.21  ULM Aβ38 p = 0.024 |
| pTau-181 | 0.9 | 0.78 | 1 | 0.75 | 0.83 | 0.92 | ULM Aβ42/40 p = 0.47  ULM Aβ42 p = 0.42  ULM Aβ40 p = 0.0009  Shim. Aβ42/40 p = 0.017  Shim. Aβ42 p = 0.024  Shim. Aβ40 p = 0.0062  Shim. Comp p = 0.19  GFAP p = 0.55  NfL p = 0.23  Simoa Aβ40 p = 0.041  Simoa Aβ42 p = 0.096  Simoa Aβ42/40 p = 0.028  ULM Aβ38 p = 0.0023 |
| Simoa Aβ40 | 0.64 | 0.41 | 0.86 | 0.35 | 0.94 | 0.42 | ULM Aβ42/40 p = 0.24  ULM Aβ42 p = 0.051  ULM Aβ40 p = 0.38  Shim. Aβ42/40 p = 0.92  Shim. Aβ42 p = 0.8  Shim. Aβ40 p = 0.41  Shim. Comp p = 0.43  GFAP p = 0.2  NfL p = 0.43  pTau-181 p = 0.041  Simoa Aβ42 p = 0.66  Simoa Aβ42/40 p = 0.71  ULM Aβ38 p = 0.48 |
| Simoa Aβ42 | 0.68 | 0.45 | 0.9 | 0.35 | 0.94 | 0.42 | ULM Aβ42/40 p = 0.28  ULM Aβ42 p = 0.12  ULM Aβ40 p = 0.25  Shim. Aβ42/40 p = 0.72  Shim. Aβ42 p = 0.92  Shim. Aβ40 p = 0.3  Shim. Comp p = 0.51  GFAP p = 0.29  NfL p = 0.6  pTau-181 p = 0.096  Simoa Aβ40 p = 0.66  Simoa Aβ42/40 p = 0.37  ULM Aβ38 p = 0.31 |
| Simoa Aβ42/40 | 0.57 | 0.33 | 0.8 | 0.29 | 0.88 | 0.42 | ULM Aβ42/40 p = 0.023  ULM Aβ42 p = 0.059  ULM Aβ40 p = 0.34  Shim. Aβ42/40 p = 0.74  Shim. Aβ42 p = 0.49  Shim. Aβ40 p = 0.98  Shim. Comp p = 0.11  GFAP p = 0.057  NfL p = 0.21  pTau-181 p = 0.028  Simoa Aβ40 p = 0.71  Simoa Aβ42 p = 0.37  ULM Aβ38 p = 0.49 |
| ULM Aβ38 | 0.48 | 0.25 | 0.71 | 0.19 | 0.61 | 0.58 | ULM Aβ42/40 p = 0.0031  ULM Aβ42 p = 0.038  ULM Aβ40 p = 0.54  Shim. Aβ42/40 p = 0.1  Shim. Aβ42 p = 0.24  Shim. Aβ40 p = 0.69  Shim. Comp p = 0.0097  GFAP p = 0.0016  NfL p = 0.024  pTau-181 p = 0.0023  Simoa Aβ40 p = 0.48  Simoa Aβ42 p = 0.31  Simoa Aβ42/40 p = 0.49 |

**Supplementary table 2:** ROC analysis bvFTD vs SCD

| Biomarker | AUC | AUC  Lower_CI | AUC  Upper_CI | Youden  Index | Sensitivity | Specificity | DeLong_Comparisons |
| --- | --- | --- | --- | --- | --- | --- | --- |
| ULM Aβ42/40 | 0.79 | 0.63 | 0.95 | 0.52 | 0.6 | 0.92 | ULM Aβ42 p = 0.78  ULM Aβ40 p = 0.0030  Shim. Aβ42/40 p = 0.0049  Shim. Aβ42 p = 0.67  Shim. Aβ40 p = 0.13  Shim. Comp p = 0.31  GFAP p = 0.016  NfL p = 0.34  pTau-181 p = 0.24  Simoa Aβ40 p = 0.55  Simoa Aβ42 p = 0.11  Simoa Aβ42/40 p = 0.38  ULM Aβ38 p = 0.012 |
| ULM Aβ42 | 0.77 | 0.6 | 0.93 | 0.52 | 0.6 | 0.92 | ULM Aβ42/40 p = 0.78  ULM Aβ40 p = 0.051  Shim. Aβ42/40 p = 0.0007  Shim. Aβ42 p = 0.78  Shim. Aβ40 p = 0.031  Shim. Comp p = 0.47  GFAP p = 0.01  NfL p = 0.28  pTau-181 p = 0.28  Simoa Aβ40 p = 0.65  Simoa Aβ42 p = 0.075  Simoa Aβ42/40 p = 0.51  ULM Aβ38 p = 0.084 |
| ULM Aβ40 | 0.41 | 0.2 | 0.63 | 0.05 | 0.55 | 0.5 | ULM Aβ42/40 p = 0.0030  ULM Aβ42 p = 0.051  Shim. Aβ42/40 p = 0.9  Shim. Aβ42 p = 0.054  Shim. Aβ40 p = 0.38  Shim. Comp p = 0.047  GFAP p = 0.74  NfL p = 0.0000  pTau-181 p = 0.2  Simoa Aβ40 p = 0.13  Simoa Aβ42 p = 0.42  Simoa Aβ42/40 p = 0.16  ULM Aβ38 p = 0.41 |
| Shim. Aβ42/40 | 0.39 | 0.18 | 0.6 | 0.083 | 0.5 | 0.58 | ULM Aβ42/40 p = 0.0049  ULM Aβ42 p = 0.0007  ULM Aβ40 p = 0.9  Shim. Aβ42 p = 0.017  Shim. Aβ40 p = 0.02  Shim. Comp p = 0.13  GFAP p = 0.49  NfL p = 0.0004  pTau-181 p = 0.071  Simoa Aβ40 p = 0.0005  Simoa Aβ42 p = 0.099  Simoa Aβ42/40 p = 0.049  ULM Aβ38 p = 0.72 |
| Shim. Aβ42 | 0.74 | 0.56 | 0.93 | 0.5 | 0.75 | 0.75 | ULM Aβ42/40 p = 0.67  ULM Aβ42 p = 0.78  ULM Aβ40 p = 0.054  Shim. Aβ42/40 p = 0.017  Shim. Aβ40 p = 0.12  Shim. Comp p = 0.45  GFAP p = 0.024  NfL p = 0.17  pTau-181 p = 0.41  Simoa Aβ40 p = 0.97  Simoa Aβ42 p = 0.26  Simoa Aβ42/40 p = 0.68  ULM Aβ38 p = 0.11 |
| Shim. Aβ40 | 0.59 | 0.38 | 0.8 | 0.23 | 0.65 | 0.58 | ULM Aβ42/40 p = 0.13  ULM Aβ42 p = 0.031  ULM Aβ40 p = 0.38  Shim. Aβ42/40 p = 0.02  Shim. Aβ42 p = 0.12  Shim. Comp p = 0.61  GFAP p = 0.35  NfL p = 0.029  pTau-181 p = 0.84  Simoa Aβ40 p = 0.067  Simoa Aβ42 p = 0.84  Simoa Aβ42/40 p = 0.51  ULM Aβ38 p = 0.49 |
| Shim. Comp | 0.67 | 0.48 | 0.86 | 0.35 | 0.6 | 0.75 | ULM Aβ42/40 p = 0.31  ULM Aβ42 p = 0.47  ULM Aβ40 p = 0.047  Shim. Aβ42/40 p = 0.13  Shim. Aβ42 p = 0.45  Shim. Aβ40 p = 0.61  GFAP p = 0.22  NfL p = 0.027  pTau-181 p = 0.75  Simoa Aβ40 p = 0.65  Simoa Aβ42 p = 0.72  Simoa Aβ42/40 p = 0.91  ULM Aβ38 p = 0.13 |
| GFAP | 0.48 | 0.25 | 0.7 | 0.17 | 0.75 | 0.42 | ULM Aβ42/40 p = 0.016  ULM Aβ42 p = 0.01  ULM Aβ40 p = 0.74  Shim. Aβ42/40 p = 0.49  Shim. Aβ42 p = 0.024  Shim. Aβ40 p = 0.35  Shim. Comp p = 0.22  NfL p = 0.0065  pTau-181 p = 0.31  Simoa Aβ40 p = 0.027  Simoa Aβ42 p = 0.34  Simoa Aβ42/40 p = 0.2  ULM Aβ38 p = 0.91 |
| NfL | 0.88 | 0.77 | 1 | 0.6 | 0.6 | 1 | ULM Aβ42/40 p = 0.34  ULM Aβ42 p = 0.28  ULM Aβ40 p = 0.0000  Shim. Aβ42/40 p = 0.0004  Shim. Aβ42 p = 0.17  Shim. Aβ40 p = 0.029  Shim. Comp p = 0.027  GFAP p = 0.0065  pTau-181 p = 0.061  Simoa Aβ40 p = 0.21  Simoa Aβ42 p = 0.046  Simoa Aβ42/40 p = 0.077  ULM Aβ38 p = 0.0004 |
| pTau-181 | 0.62 | 0.41 | 0.83 | 0.38 | 0.55 | 0.83 | ULM Aβ42/40 p = 0.24  ULM Aβ42 p = 0.28  ULM Aβ40 p = 0.2  Shim. Aβ42/40 p = 0.071  Shim. Aβ42 p = 0.41  Shim. Aβ40 p = 0.84  Shim. Comp p = 0.75  GFAP p = 0.31  NfL p = 0.061  Simoa Aβ40 p = 0.44  Simoa Aβ42 p = 0.84  Simoa Aβ42/40 p = 0.74  ULM Aβ38 p = 0.3 |
| Simoa Aβ40 | 0.72 | 0.54 | 0.91 | 0.38 | 0.63 | 0.75 | ULM Aβ42/40 p = 0.55  ULM Aβ42 p = 0.65  ULM Aβ40 p = 0.13  Shim. Aβ42/40 p = 0.0005  Shim. Aβ42 p = 0.97  Shim. Aβ40 p = 0.067  Shim. Comp p = 0.65  GFAP p = 0.027  NfL p = 0.21  pTau-181 p = 0.44  Simoa Aβ42 p = 0.12  Simoa Aβ42/40 p = 0.62  ULM Aβ38 p = 0.2 |
| Simoa Aβ42 | 0.6 | 0.37 | 0.82 | 0.28 | 0.53 | 0.75 | ULM Aβ42/40 p = 0.11  ULM Aβ42 p = 0.075  ULM Aβ40 p = 0.42  Shim. Aβ42/40 p = 0.099  Shim. Aβ42 p = 0.26  Shim. Aβ40 p = 0.84  Shim. Comp p = 0.72  GFAP p = 0.34  NfL p = 0.046  pTau-181 p = 0.84  Simoa Aβ40 p = 0.12  Simoa Aβ42/40 p = 0.67  ULM Aβ38 p = 0.56 |
| Simoa Aβ42/40 | 0.67 | 0.47 | 0.87 | 0.34 | 0.42 | 0.92 | ULM Aβ42/40 p = 0.38  ULM Aβ42 p = 0.51  ULM Aβ40 p = 0.16  Shim. Aβ42/40 p = 0.049  Shim. Aβ42 p = 0.68  Shim. Aβ40 p = 0.51  Shim. Comp p = 0.91  GFAP p = 0.2  NfL p = 0.077  pTau-181 p = 0.74  Simoa Aβ40 p = 0.62  Simoa Aβ42 p = 0.67  ULM Aβ38 p = 0.26 |
| ULM Aβ38 | 0.45 | 0.23 | 0.68 | 0.1 | 0.85 | 0.25 | ULM Aβ42/40 p = 0.012  ULM Aβ42 p = 0.084  ULM Aβ40 p = 0.41  Shim. Aβ42/40 p = 0.72  Shim. Aβ42 p = 0.11  Shim. Aβ40 p = 0.49  Shim. Comp p = 0.13  GFAP p = 0.91  NfL p = 0.0004  pTau-181 p = 0.3  Simoa Aβ40 p = 0.2  Simoa Aβ42 p = 0.56  Simoa Aβ42/40 p = 0.26 |

**Supplementary table 3:** ROC analysis AD vs bvFTD

| Biomarker | AUC | AUC  Lower_CI | AUC  Upper_CI | Youden  Index | Sensitivity | Specificity | DeLong_Comparisons |
| --- | --- | --- | --- | --- | --- | --- | --- |
| ULM Aβ42/40 | 0.44 | 0.25 | 0.63 | 0.061 | 0.11 | 0.95 | ULM Aβ42 p = 0.5  ULM Aβ40 p = 0.5  Shim. Aβ42/40 p = 0.52  Shim. Aβ42 p = 0.67  Shim. Aβ40 p = 0.67  Shim. Comp p = 0.19  GFAP p = 0.0001  NfL p = 0.23  pTau-181 p = 0.0000  Simoa Aβ40 p = 0.31  Simoa Aβ42 p = 0.44  Simoa Aβ42/40 p = 0.072  ULM Aβ38 p = 0.53 |
| ULM Aβ42 | 0.52 | 0.33 | 0.71 | 0.18 | 0.83 | 0.35 | ULM Aβ42/40 p = 0.5  ULM Aβ40 p = 0.67  Shim. Aβ42/40 p = 0.86  Shim. Aβ42 p = 0.92  Shim. Aβ40 p = 0.91  Shim. Comp p = 0.44  GFAP p = 0.0011  NfL p = 0.49  pTau-181 p = 0.0012  Simoa Aβ40 p = 0.27  Simoa Aβ42 p = 0.7  Simoa Aβ42/40 p = 0.064  ULM Aβ38 p = 0.79 |
| ULM Aβ40 | 0.55 | 0.36 | 0.74 | 0.25 | 1 | 0.25 | ULM Aβ42/40 p = 0.5  ULM Aβ42 p = 0.67  Shim. Aβ42/40 p = 0.96  Shim. Aβ42 p = 0.78  Shim. Aβ40 p = 0.6  Shim. Comp p = 0.55  GFAP p = 0.0031  NfL p = 0.59  pTau-181 p = 0.0046  Simoa Aβ40 p = 0.18  Simoa Aβ42 p = 0.8  Simoa Aβ42/40 p = 0.025  ULM Aβ38 p = 0.93 |
| Shim. Aβ42/40 | 0.54 | 0.35 | 0.73 | 0.22 | 0.72 | 0.5 | ULM Aβ42/40 p = 0.52  ULM Aβ42 p = 0.86  ULM Aβ40 p = 0.96  Shim. Aβ42 p = 0.73  Shim. Aβ40 p = 0.71  Shim. Comp p = 0.26  GFAP p = 0.0022  NfL p = 0.52  pTau-181 p = 0.0014  Simoa Aβ40 p = 0.42  Simoa Aβ42 p = 0.85  Simoa Aβ42/40 p = 0.035  ULM Aβ38 p = 1 |
| Shim. Aβ42 | 0.5 | 0.31 | 0.69 | 0.16 | 0.56 | 0.6 | ULM Aβ42/40 p = 0.67  ULM Aβ42 p = 0.92  ULM Aβ40 p = 0.78  Shim. Aβ42/40 p = 0.73  Shim. Aβ40 p = 0.98  Shim. Comp p = 0.15  GFAP p = 0.0011  NfL p = 0.32  pTau-181 p = 0.0001  Simoa Aβ40 p = 0.53  Simoa Aβ42 p = 0.59  Simoa Aβ42/40 p = 0.046  ULM Aβ38 p = 0.79 |
| Shim. Aβ40 | 0.51 | 0.31 | 0.7 | 0.21 | 0.56 | 0.65 | ULM Aβ42/40 p = 0.67  ULM Aβ42 p = 0.91  ULM Aβ40 p = 0.6  Shim. Aβ42/40 p = 0.71  Shim. Aβ42 p = 0.98  Shim. Comp p = 0.36  GFAP p = 0.0011  NfL p = 0.39  pTau-181 p = 0.0012  Simoa Aβ40 p = 0.12  Simoa Aβ42 p = 0.65  Simoa Aβ42/40 p = 0.027  ULM Aβ38 p = 0.66 |
| Shim. Comp | 0.62 | 0.44 | 0.81 | 0.31 | 0.61 | 0.7 | ULM Aβ42/40 p = 0.19  ULM Aβ42 p = 0.44  ULM Aβ40 p = 0.55  Shim. Aβ42/40 p = 0.26  Shim. Aβ42 p = 0.15  Shim. Aβ40 p = 0.36  GFAP p = 0.025  NfL p = 1  pTau-181 p = 0.0072  Simoa Aβ40 p = 0.91  Simoa Aβ42 p = 0.64  Simoa Aβ42/40 p = 0.2  ULM Aβ38 p = 0.49 |
| GFAP | 0.87 | 0.76 | 0.98 | 0.62 | 0.67 | 0.95 | ULM Aβ42/40 p = 0.0001  ULM Aβ42 p = 0.0011  ULM Aβ40 p = 0.0031  Shim. Aβ42/40 p = 0.0022  Shim. Aβ42 p = 0.0011  Shim. Aβ40 p = 0.0011  Shim. Comp p = 0.025  NfL p = 0.056  pTau-181 p = 0.71  Simoa Aβ40 p = 0.023  Simoa Aβ42 p = 0.025  Simoa Aβ42/40 p = 0.38  ULM Aβ38 p = 0.0036 |
| NfL | 0.62 | 0.44 | 0.81 | 0.38 | 0.83 | 0.55 | ULM Aβ42/40 p = 0.23  ULM Aβ42 p = 0.49  ULM Aβ40 p = 0.59  Shim. Aβ42/40 p = 0.52  Shim. Aβ42 p = 0.32  Shim. Aβ40 p = 0.39  Shim. Comp p = 1  GFAP p = 0.056  pTau-181 p = 0.013  Simoa Aβ40 p = 0.93  Simoa Aβ42 p = 0.56  Simoa Aβ42/40 p = 0.26  ULM Aβ38 p = 0.55 |
| pTau-181 | 0.9 | 0.79 | 1 | 0.79 | 0.89 | 0.9 | ULM Aβ42/40 p = 0.0000  ULM Aβ42 p = 0.0012  ULM Aβ40 p = 0.0046  Shim. Aβ42/40 p = 0.0014  Shim. Aβ42 p = 0.0001  Shim. Aβ40 p = 0.0012  Shim. Comp p = 0.0072  GFAP p = 0.71  NfL p = 0.013  Simoa Aβ40 p = 0.02  Simoa Aβ42 p = 0.0035  Simoa Aβ42/40 p = 0.23  ULM Aβ38 p = 0.0039 |
| Simoa Aβ40 | 0.61 | 0.42 | 0.81 | 0.3 | 0.88 | 0.42 | ULM Aβ42/40 p = 0.31  ULM Aβ42 p = 0.27  ULM Aβ40 p = 0.18  Shim. Aβ42/40 p = 0.42  Shim. Aβ42 p = 0.53  Shim. Aβ40 p = 0.12  Shim. Comp p = 0.91  GFAP p = 0.023  NfL p = 0.93  pTau-181 p = 0.02  Simoa Aβ42 p = 0.78  Simoa Aβ42/40 p = 0.085  ULM Aβ38 p = 0.28 |
| Simoa Aβ42 | 0.56 | 0.37 | 0.76 | 0.26 | 1 | 0.26 | ULM Aβ42/40 p = 0.44  ULM Aβ42 p = 0.7  ULM Aβ40 p = 0.8  Shim. Aβ42/40 p = 0.85  Shim. Aβ42 p = 0.59  Shim. Aβ40 p = 0.65  Shim. Comp p = 0.64  GFAP p = 0.025  NfL p = 0.56  pTau-181 p = 0.0035  Simoa Aβ40 p = 0.78  Simoa Aβ42/40 p = 0.11  ULM Aβ38 p = 0.8 |
| Simoa Aβ42/40 | 0.76 | 0.58 | 0.93 | 0.61 | 0.88 | 0.74 | ULM Aβ42/40 p = 0.072  ULM Aβ42 p = 0.064  ULM Aβ40 p = 0.025  Shim. Aβ42/40 p = 0.035  Shim. Aβ42 p = 0.046  Shim. Aβ40 p = 0.027  Shim. Comp p = 0.2  GFAP p = 0.38  NfL p = 0.26  pTau-181 p = 0.23  Simoa Aβ40 p = 0.085  Simoa Aβ42 p = 0.11  ULM Aβ38 p = 0.025 |
| ULM Aβ38 | 0.54 | 0.35 | 0.73 | 0.21 | 0.61 | 0.6 | ULM Aβ42/40 p = 0.53  ULM Aβ42 p = 0.79  ULM Aβ40 p = 0.93  Shim. Aβ42/40 p = 1  Shim. Aβ42 p = 0.79  Shim. Aβ40 p = 0.66  Shim. Comp p = 0.49  GFAP p = 0.0036  NfL p = 0.55  pTau-181 p = 0.0039  Simoa Aβ40 p = 0.28  Simoa Aβ42 p = 0.8  Simoa Aβ42/40 p = 0.025 |
